# 575 Single-stage Trilaminar Skin Reconstruction Following Full-Thickness Burn Injury

**DOI:** 10.1093/jbcr/irad045.170

**Published:** 2023-05-15

**Authors:** Fuat Baris Bengur, Shawn J Loder, Yadira Villalvazo, Pooja Humar, Wayne V Nerone, Phoebe L Lee, Lauren E Kokai, Kacey G Marra, Elof Eriksson, J Peter Rubin

**Affiliations:** University of Pittsburgh Department of Plastic Surgery, Pittsburgh, Pennsylvania; University of Pittsburgh Department of Plastic Surgery, Pittsburgh, Pennsylvania; University of Pittsburgh Department of Plastic Surgery, Pittsburgh, Pennsylvania; University of Pittsburgh Department of Plastic Surgery, Pittsburgh, Pennsylvania; University of Pittsburgh Department of Plastic Surgery, Pittsburgh, Pennsylvania; University of Pittsburgh Department of Plastic Surgery, Pittsburgh, Pennsylvania; Unviersity of Pittsburgh Department of Plastic Surgery, Pittsburgh, Pennsylvania; University of Pittsburgh Department of Plastic Surgery, Pittsburgh, Pennsylvania; Harvard Medical School, Boston, Massachusetts; University of Pittsburgh Department of Plastic Surgery, Pittsburgh, Pennsylvania; University of Pittsburgh Department of Plastic Surgery, Pittsburgh, Pennsylvania; University of Pittsburgh Department of Plastic Surgery, Pittsburgh, Pennsylvania; University of Pittsburgh Department of Plastic Surgery, Pittsburgh, Pennsylvania; University of Pittsburgh Department of Plastic Surgery, Pittsburgh, Pennsylvania; University of Pittsburgh Department of Plastic Surgery, Pittsburgh, Pennsylvania; University of Pittsburgh Department of Plastic Surgery, Pittsburgh, Pennsylvania; Unviersity of Pittsburgh Department of Plastic Surgery, Pittsburgh, Pennsylvania; University of Pittsburgh Department of Plastic Surgery, Pittsburgh, Pennsylvania; Harvard Medical School, Boston, Massachusetts; University of Pittsburgh Department of Plastic Surgery, Pittsburgh, Pennsylvania; University of Pittsburgh Department of Plastic Surgery, Pittsburgh, Pennsylvania; University of Pittsburgh Department of Plastic Surgery, Pittsburgh, Pennsylvania; University of Pittsburgh Department of Plastic Surgery, Pittsburgh, Pennsylvania; University of Pittsburgh Department of Plastic Surgery, Pittsburgh, Pennsylvania; University of Pittsburgh Department of Plastic Surgery, Pittsburgh, Pennsylvania; University of Pittsburgh Department of Plastic Surgery, Pittsburgh, Pennsylvania; Unviersity of Pittsburgh Department of Plastic Surgery, Pittsburgh, Pennsylvania; University of Pittsburgh Department of Plastic Surgery, Pittsburgh, Pennsylvania; Harvard Medical School, Boston, Massachusetts; University of Pittsburgh Department of Plastic Surgery, Pittsburgh, Pennsylvania; University of Pittsburgh Department of Plastic Surgery, Pittsburgh, Pennsylvania; University of Pittsburgh Department of Plastic Surgery, Pittsburgh, Pennsylvania; University of Pittsburgh Department of Plastic Surgery, Pittsburgh, Pennsylvania; University of Pittsburgh Department of Plastic Surgery, Pittsburgh, Pennsylvania; University of Pittsburgh Department of Plastic Surgery, Pittsburgh, Pennsylvania; University of Pittsburgh Department of Plastic Surgery, Pittsburgh, Pennsylvania; Unviersity of Pittsburgh Department of Plastic Surgery, Pittsburgh, Pennsylvania; University of Pittsburgh Department of Plastic Surgery, Pittsburgh, Pennsylvania; Harvard Medical School, Boston, Massachusetts; University of Pittsburgh Department of Plastic Surgery, Pittsburgh, Pennsylvania; University of Pittsburgh Department of Plastic Surgery, Pittsburgh, Pennsylvania; University of Pittsburgh Department of Plastic Surgery, Pittsburgh, Pennsylvania; University of Pittsburgh Department of Plastic Surgery, Pittsburgh, Pennsylvania; University of Pittsburgh Department of Plastic Surgery, Pittsburgh, Pennsylvania; University of Pittsburgh Department of Plastic Surgery, Pittsburgh, Pennsylvania; University of Pittsburgh Department of Plastic Surgery, Pittsburgh, Pennsylvania; Unviersity of Pittsburgh Department of Plastic Surgery, Pittsburgh, Pennsylvania; University of Pittsburgh Department of Plastic Surgery, Pittsburgh, Pennsylvania; Harvard Medical School, Boston, Massachusetts; University of Pittsburgh Department of Plastic Surgery, Pittsburgh, Pennsylvania; University of Pittsburgh Department of Plastic Surgery, Pittsburgh, Pennsylvania; University of Pittsburgh Department of Plastic Surgery, Pittsburgh, Pennsylvania; University of Pittsburgh Department of Plastic Surgery, Pittsburgh, Pennsylvania; University of Pittsburgh Department of Plastic Surgery, Pittsburgh, Pennsylvania; University of Pittsburgh Department of Plastic Surgery, Pittsburgh, Pennsylvania; University of Pittsburgh Department of Plastic Surgery, Pittsburgh, Pennsylvania; Unviersity of Pittsburgh Department of Plastic Surgery, Pittsburgh, Pennsylvania; University of Pittsburgh Department of Plastic Surgery, Pittsburgh, Pennsylvania; Harvard Medical School, Boston, Massachusetts; University of Pittsburgh Department of Plastic Surgery, Pittsburgh, Pennsylvania; University of Pittsburgh Department of Plastic Surgery, Pittsburgh, Pennsylvania; University of Pittsburgh Department of Plastic Surgery, Pittsburgh, Pennsylvania; University of Pittsburgh Department of Plastic Surgery, Pittsburgh, Pennsylvania; University of Pittsburgh Department of Plastic Surgery, Pittsburgh, Pennsylvania; University of Pittsburgh Department of Plastic Surgery, Pittsburgh, Pennsylvania; University of Pittsburgh Department of Plastic Surgery, Pittsburgh, Pennsylvania; Unviersity of Pittsburgh Department of Plastic Surgery, Pittsburgh, Pennsylvania; University of Pittsburgh Department of Plastic Surgery, Pittsburgh, Pennsylvania; Harvard Medical School, Boston, Massachusetts; University of Pittsburgh Department of Plastic Surgery, Pittsburgh, Pennsylvania; University of Pittsburgh Department of Plastic Surgery, Pittsburgh, Pennsylvania; University of Pittsburgh Department of Plastic Surgery, Pittsburgh, Pennsylvania; University of Pittsburgh Department of Plastic Surgery, Pittsburgh, Pennsylvania; University of Pittsburgh Department of Plastic Surgery, Pittsburgh, Pennsylvania; University of Pittsburgh Department of Plastic Surgery, Pittsburgh, Pennsylvania; University of Pittsburgh Department of Plastic Surgery, Pittsburgh, Pennsylvania; Unviersity of Pittsburgh Department of Plastic Surgery, Pittsburgh, Pennsylvania; University of Pittsburgh Department of Plastic Surgery, Pittsburgh, Pennsylvania; Harvard Medical School, Boston, Massachusetts; University of Pittsburgh Department of Plastic Surgery, Pittsburgh, Pennsylvania; University of Pittsburgh Department of Plastic Surgery, Pittsburgh, Pennsylvania; University of Pittsburgh Department of Plastic Surgery, Pittsburgh, Pennsylvania; University of Pittsburgh Department of Plastic Surgery, Pittsburgh, Pennsylvania; University of Pittsburgh Department of Plastic Surgery, Pittsburgh, Pennsylvania; University of Pittsburgh Department of Plastic Surgery, Pittsburgh, Pennsylvania; University of Pittsburgh Department of Plastic Surgery, Pittsburgh, Pennsylvania; Unviersity of Pittsburgh Department of Plastic Surgery, Pittsburgh, Pennsylvania; University of Pittsburgh Department of Plastic Surgery, Pittsburgh, Pennsylvania; Harvard Medical School, Boston, Massachusetts; University of Pittsburgh Department of Plastic Surgery, Pittsburgh, Pennsylvania; University of Pittsburgh Department of Plastic Surgery, Pittsburgh, Pennsylvania; University of Pittsburgh Department of Plastic Surgery, Pittsburgh, Pennsylvania; University of Pittsburgh Department of Plastic Surgery, Pittsburgh, Pennsylvania; University of Pittsburgh Department of Plastic Surgery, Pittsburgh, Pennsylvania; University of Pittsburgh Department of Plastic Surgery, Pittsburgh, Pennsylvania; University of Pittsburgh Department of Plastic Surgery, Pittsburgh, Pennsylvania; Unviersity of Pittsburgh Department of Plastic Surgery, Pittsburgh, Pennsylvania; University of Pittsburgh Department of Plastic Surgery, Pittsburgh, Pennsylvania; Harvard Medical School, Boston, Massachusetts; University of Pittsburgh Department of Plastic Surgery, Pittsburgh, Pennsylvania

## Abstract

**Introduction:**

Complex burns are highly morbid injuries that can cause severe disfigurement and can be devastating to one’s quality of life and psychosocial well-being. Currently, there are no simple, single-stage procedures available for extensive or multifocal burns to address full-thickness trilaminar defects. Our team has previously demonstrated the viability of an adipose-first reconstruction to address hypodermal defects. In this study, we demonstrate the efficacy of a combined adipose plus finely minced skin to achieve a single-stage trilaminar skin reconstruction.

**Methods:**

Full-thickness burns were created on female Yorkshire swine. After 48-hours, escharectomies were performed to the level of fascia. The wounds were layered with adipose harvested from female Yorkshire swine. In one group, autologous split-thickness skin grafts were cut into pixel size (0.3x0.3 mm) grafts and layered on top of the adipose. Pigs were maintained for 4-weeks with weekly photography, ultrasound, followed by endpoint histology and tension measurements.

**Results:**

At the end of the 4-week period, adipose combined with pixel graft demonstrated improved epithelialization and less contracture (p< 0.01). Thickness and mobility measurements were consistent in both groups. This findings were similar to our previous approaches using adipose following surgical debridement. Tissue pliability in the pixel grafting group was maintained to a high degree. Cross sections were performed which showed the persistence of fat graft at the base of the wounds.

**Conclusions:**

Immediate, single-stage trilaminar reconstruction of full-thickness complex burns reduces contracture, mitigates adhesion, and restores normal soft-tissue thickness, therefore, presenting a paradigm changing approach in the current practice of burn injuries to the mobile surfaces.

**Applicability of Research to Practice:**

Our results support the therapeutic potential of adipose-based soft-tissue reconstruction of burns to both enhance hypodermal augmentation and mitigate fibrotic complications including contracture and adhesions. Our data suggest that this approach is compatible with the standard of care skin grafting, and in fact provides improved epidermal thickness and pliability.

